# Fatal airway obstruction due to endobronchial bleeding post cryobiopsy: a case report

**DOI:** 10.1093/jscr/rjaf231

**Published:** 2025-04-19

**Authors:** Muhammad Nadzir, Isnin Anang Marhana, Farah Fatmawati

**Affiliations:** Department of Pulmonology and Respiratory Medicine, Faculty of Medicine, Airlangga University – Dr. Soetomo General Academic Hospital, Jl. Mayjen Prof. Dr. Moesto po No. 6-8, Surabaya 60286, Indonesia; Department of Pulmonology and Respiratory Medicine, Faculty of Medicine, Airlangga University – Dr. Soetomo General Academic Hospital, Jl. Mayjen Prof. Dr. Moesto po No. 6-8, Surabaya 60286, Indonesia; Department of Pulmonology and Respiratory Medicine, Faculty of Medicine, Airlangga University – Dr. Soetomo General Academic Hospital, Jl. Mayjen Prof. Dr. Moesto po No. 6-8, Surabaya 60286, Indonesia

**Keywords:** bleeding, cryobiopsy, obstruction

## Abstract

Cryobiopsy offers an advantage because of the extraction of more extensive tissue and enhanced diagnostic accuracy. However, the risk of bleeding from the biopsy site is higher. We report a lung tumor patient who experienced bleeding in the right main bronchus after cryobiopsy. Suctioning was performed, cold saline was administered, and the bleeding stopped. Five minutes after the procedure, the patient experienced desaturation. A re-bronchoscopy found massive bleeding in the right main bronchus which spread to the left main bronchus. Suctioning was performed, cold saline and epinephrine were administered. Before further treatment was given, the patient got cardiac arrest and he was treated in the intensive care unit. This highlighted the importance of training and considering the additional precautions for bleeding control. Intensive monitoring of the patients is needed when performing cryobiopsy to prepare for the early management of complications, especially for those patients with risk factors for bleeding in cryobiopsy.

## Introduction

Lung cancer is the leading cause of cancer-related deaths worldwide. In order to improve the prognosis of lung cancer patients, a complete characterization of the tumor needs to performed, with the determination of all the molecular alterations that can be targeted by novel therapies. Therefore, using a biopsy method that provides safe and adequate lung tissue sampling, without morphological alterations, such as cryobiopsy, could provide a better choice for these patients [1].

Cryobiopsy is one of the methods to manage endobronchial mass in lung cancer. The main advantage of cryobiopsy is its ability to extract larger tissue specimens with fewer crushing artifacts, which improves diagnostic accuracy [2]. As cryobiopsy samples tend to be larger, the risk of bleeding from the biopsy site is higher and occasionally causes moderate or severe bleeding [3]. The primary concerns regarding lung cryobiopsy are patient safety, with bleeding being the most feared complication and may be life threatening [4, 5]. Complications following cryobiopsy include bleeding (6%–70%) and pneumothorax (0%–20%). Diagnostic accuracy is about 86%, with low mortality (0%–3%). The risk of hypoxemia and asphyxia increases with massive bleeding, particularly when the contralateral bronchial tree is affected, possibly requiring prolonged ventilation [2]. The concerns regarding procedure related complications in lung cancer patients, such as bleeding and pneumothorax, need to be properly addressed [1].

## Case report

A 62-year-old male from Indonesia complained of experiencing shortness of breath, especially during heavy activities, for the past month. The patient has a smoking history of 12 cigarettes per day over the last 40 years and no reported history of hypertension, diabetes, or prior tuberculosis infection.

**Figure 1:**
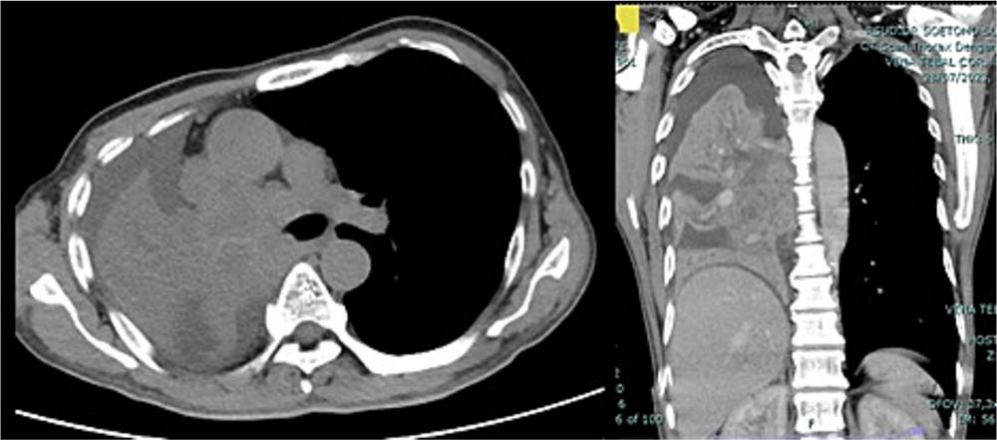
CT scan thorax with contrast showed enhancing solid mass measuring ±9.6 × 9.5 × 14.5 cm in the centre of the right lung extending to the entire right lung, middle, and subcarinal mediastinum mass.

The patient's general condition is good. Laboratory findings indicated normal levels of hemoglobin (12.6 g/dl), platelets (447 000/μl), prothrombin time (14.9 s), and activated partial thromboplastin time (34.9 s). CT scan results showed an enhancing solid mass of the right lung extending to the entire right lung (Fig. 1). The patient was diagnosed with right lung cancer T4N2M1a stage IVA with right lung atelectasis.

**Figure 2:**
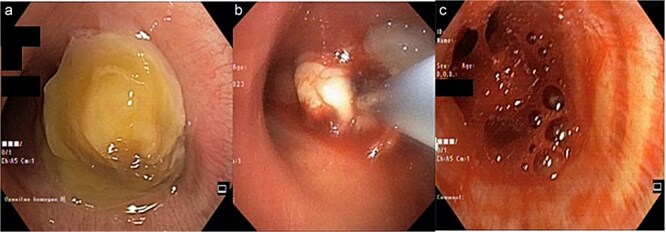
(a) Mass at the proximal of the right main bronchus, left main bronchus was still patent; (b) a cryobiopsy was performed on an intraluminal mass in the right main bronchus; (c) bleeding 5 minutes after the cryobiopsy was done which extended to the left main bronchus so it covered both main bronchus.

Bronchoscopy results (Fig. 2) showed that an intraluminal mass entirely covered the proximal lumen of the right main bronchus. A cryobiopsy was performed on the mass in the proximal lumen. After a cryobiopsy, bleeding occurred in the right main bronchus. Suctioning was performed, cold saline was administered, and then the bleeding stopped. The bronchoscope was withdrawn, and the procedure was completed. Five minutes after the procedure the patient experienced desaturation. A re-bronchoscopy was performed for re-evaluation and found massive bleeding in the right main bronchus which spread to the left main bronchus (Fig. 2). Suctioning was performed, cold saline and epinephrine were administered. Not long after that, the patient experienced cardiac arrest. CPR was performed, and the patient got the return of spontaneous circulation so he was transferred to the ICU. Three days after treatment in ICU the patient passed away due to acute respiratory failure.

## Discussion

Complications of cryobiopsy include mild, moderate to severe bleeding, pneumothorax, need for prolonged mechanical ventilation, prolonged hypoxemia, post-procedure infection or fever, and death. Other rare complications include pneumomediastinum and subcutaneous emphysema [2, 6]. A previous study also reported mild bleeding (31.9%) and moderate bleeding (8.5%) in cryobiopsy [7]. Moderate bleeding occurred after cryobiopsy in two patients and was managed by argon plasma coagulation [8]. Another study showed an increased risk of bleeding when ≥ cryobiopsies were obtained (OR = 2.758) [9].

According to the European Respiratory Society, the risk of bleeding complications during bronchoscopy biopsy is influenced by several factors. Based on the type of procedure performed, BAL inspection and examination is the procedure with the lowest complications and cryobiopsy is the procedure with the most bleeding complications. This is because cryobiopsy takes large amounts of tissue, causing increased bleeding complications, so this procedure usually involves placing a tampon with a balloon occluder in the segment where the biopsy is being performed [10]. The patient in this report had stage 4 cancer with an endobronchial mass in the right main bronchus, which indicates that it is at an advanced stage and the mass is located in the central airway, which increases the risk of bleeding during the endobronchial biopsy. After the cryobiopsy was carried out, the bleeding appeared to be increasingly massive and began to spread to the left main bronchus. This was in accordance with the literature which shows that cryobiopsy is a procedure with the highest risk.

In this case report, the cryobiopsy was chosen because it offers an advantage because of the extraction of more extensive tissue and enhanced diagnostic accuracy. The main advantage of cryobiopsy is its ability to extract larger tissue specimens with fewer crushing artifacts, which improves diagnostic accuracy [2]. However, the risk of bleeding from the biopsy site is higher and occasionally causes moderate or severe bleeding as cryobiopsy samples tend to be larger [3]. Therefore, the prevention and management the risk of bleeding have been prepared, including the preparation of tampon balloons. Suction, cold saline solution administration, and adrenaline were also applied for the management of initial endobronchial bleeding.

As cryobiopsy samples tend to be larger, the risk of bleeding from the biopsy site is higher and occasionally causes moderate or severe bleeding [3]. Furthermore, some risk factors also contribute in the incidence of the bleeding when performing cryobiopsy. A previous study reported that the ground-glass feature of the lesion was a significant factor for clinically significant bleeding. Sample size over 15 mm^2^ also tended to have a higher bleeding rate. In multivariable analysis, the ground glass feature of the lesion had a significant effect on the clinically significant bleeding rate compared to a solid feature [11].

Initial endobronchial bleeding management includes suction, cold saline solution administration, and adrenaline. Management of endobronchial hemostasis is based on the grade of bleeding, where 50% of patients only require intrabronchial administration of 5–10 ml of cold saline (2–3 times repeated if necessary) to stop bleeding, while the remaining patients require administration of small amounts of topical adrenaline (1:10 000) [12]. The use of various types of tampon balloons helps isolate bleeding. Fogarty balloons have many advantages as they are globally available and come in various sizes. Another specially developed tamponade balloon is a double-lumen bronchial barrier catheter balloon that can be inserted through a flexible bronchoscopy tube for placement into the bleeding bronchial segment. In addition, two lumens allow the entry of substances that improve hemostasis. Successful use of this balloon has been reported in 26 patients with moderate to severe bleeding due to different conditions [13].

A bronchial balloon blocker has been prophylactically used to prevent flooding of blood into the central airway when performing cryobiopsy. Although the balloon occlusion method is effective to avoid severe bleeding, guiding the balloon blocker to the occlusion site is sometimes challenging and complicates the cryobiopsy procedure [3]. The two-scope technique is an alternative method of hemostasis for cryobiopsy which has established safety and diagnostic utility. It allows physicians to control bleeding promptly and manipulate devices flexibly during the procedure [11]. Another study suggested that the tube-wedging method is simple and safe, and may be a promising alternative to the balloon occlusion method for transbronchial cryobiopsies [3]. Pulmonologists should be more careful, especially when carrying out cryobiopsy because the bleeding complication is higher than that of another procedure.

This case report highlighted the importance of training and to consider the additional precautions for bleeding control. Intensive monitoring of the patients is needed when performing cryobiopsy to prepare for the early management of complications, especially for those patients with risk factors for bleeding in cryobiopsy.
